# Confounding of Cerebral Blood Flow Velocity by Blood Pressure During Breath Holding or Hyperventilation in Transient Ischemic Attack or Stroke

**DOI:** 10.1161/STROKEAHA.119.027829

**Published:** 2020-01-27

**Authors:** Alastair J.S. Webb, Matteo Paolucci, Sara Mazzucco, Linxin Li, Peter M. Rothwell

**Affiliations:** 1 From the Department of Clinical Neuroscience, Wolfson Centre for Prevention of Stroke and Dementia, University of Oxford, United Kingdom (A.J.S.W., S.M., L.L., P.M.R.); 2 Headache and Neurosonology Unit, Neurology Department, Università Campus Bio-Medico, Rome, Italy (M.P.).

**Keywords:** blood pressure, humans, ischemic attack, transient, leukoaraiosis, linear models, stroke

## Abstract

Breath holding (BH) and hyperventilation are used to assess abnormal cerebrovascular reactivity, often in relation to severity of small vessel disease and risk of stroke with carotid stenosis, but responses may be confounded by blood pressure (BP) changes. We compared effects of BP and end-tidal carbon dioxide (etCO_2_) on middle cerebral artery mean flow velocity (MFV) in consecutive transient ischemic attack and minor stroke patients.

**Methods—:**

In the population-based, prospective OXVASC (Oxford Vascular Study) phenotyped cohort, change in MFV on transcranial Doppler ultrasound (ΔMFV, DWL-DopplerBox), beat-to-beat BP (Finometer), and etCO_2_ was measured during 30 seconds of BH or hyperventilation. Two blinded reviewers independently assessed recording quality. Dependence of ΔMFV on ΔBP and ΔetCO_2_ was determined by general linear models, stratified by quartiles.

**Results—:**

Four hundred eighty-eight of 602 (81%) patients with adequate bone windows had high-quality recordings, more often in younger participants (64.6 versus 68.7 years; *P*<0.01), whereas 426 had hyperventilation tests (70.7%). During BH, ΔMFV was correlated with a rise in mean blood pressure (MBP; r^2^=0.15, *P*<0.001) but not ΔCO_2_ (r^2^=0.002, *P*=0.32), except in patients with ΔMBP <10% (r^2^=0.13, *P*<0.001). In contrast during hyperventilation, the fall in MFV was similarly correlated with reduction in CO_2_ and reduction in MBP (ΔCO_2_: r^2^=0.13, *P*<0.001; ΔMBP: r^2^=0.12, *P*<0.001), with a slightly greater effect of ΔCO_2_ when ΔMBP was <10% (r^2^=0.15). Stratifying by quartile, MFV increased linearly during BH across quartiles of ΔMBP, with no increase with ΔetCO_2_. In contrast, during hyperventilation, MFV decreased linearly with ΔetCO_2_, independent of ΔMBP.

**Conclusions—:**

In older patients with recent transient ischemic attack or minor stroke, cerebral blood flow responses to BH were confounded by BP changes but reflected etCO_2_ change during hyperventilation. Correct interpretation of cerebrovascular reactivity responses to etCO_2_, including in small vessel disease and carotid stenosis, requires concurrent BP measurement.

## Abstract 2

Supplemental Digital Content is available in the text.

Maintenance of cerebral perfusion is dependent on autoregulation of systemic blood pressure (BP) and endothelium-dependent reactivity of the cerebral circulation to stimuli such as carbon dioxide (CO_2_).^1–3^ Endothelial dysfunction is emerging as a significant pathophysiological feature of cerebral small vessel disease,^4,5^ which contributes to ≤30% of strokes^6^ and 40% of dementia,^7,8^ and is independently associated with hemodynamically significant carotid stenosis^9^ and the associated risk of recurrent stroke^10,11^ and revascularization injury after carotid endarterectomy.^12^

Accurate and practical measurement of cerebrovascular reactivity (CVR) to CO_2_ is critical to understanding its pathophysiological role and potential as a treatment target. Previous reports have used a range of methods to assess CVR, demonstrating that abnormal responses to breath holding (BH) are associated with a >3-fold increased risk of recurrent stroke in patients with severe stenosis or carotid occlusion^13,14^ and a 5-fold increased risk of cognitive deterioration in mild cognitive impairment patients,^15,16^ that a 10% reduction in CVR during CO_2_ inhalation on magnetic resonance imaging is associated with a 30% to 50% increased severity of small vessel disease,^17^ and that abnormal responses to hyperventilation are linked to an increased risk of recurrent stroke in severe carotid stenosis^18^ and to vascular and degenerative dementia.^19^ However, the most valid and practical methods to assess CVR are uncertain, particularly because CVR responses are assumed to measure cerebrovascular endothelial responses to CO_2_, disregarding any contribution of changes in BP.

Therefore, we determined the feasibility, practicality, and physiological validity of BH and hyperventilation testing for assessment of CVR on transcranial Doppler ultrasound in a pragmatic, large population-based prospective cohort of patients with acute transient ischemic attack or minor stroke.

## Methods

### Study Population

Consecutive, consenting patients with transient ischemic attack or minor stroke were recruited between April 2011 and April 2018, as part of the phenotyped cohort of the OXVASC (Oxford Vascular Study).^20,21^ Participants were recruited at the OXVASC daily emergency clinic, either following a referral after attendance at the Emergency Department or after direct referral from primary care, usually within 24 hours. Patients were referred after transient neurological symptoms or symptoms consistent with a minor stroke, not requiring direct admission to hospital. The OXVASC population consists of >92 000 individuals registered with about 100 primary-care physicians in Oxfordshire, United Kingdom, with extensive clinical assessment and prospective face-to-face follow-up, as reported previously.^21,22^

As part of the OXVASC cohort, a routine prospective cardiovascular physiological assessment is performed at the 1-month visit, including a BH assessment of CVR since April 2011 and hyperventilation since September 2011. Participants were excluded if they were <18 years of age, cognitively impaired (The Mini-Mental State Exam <23), pregnant, had active cancer, autonomic failure, a recent myocardial infarction, unstable angina, heart failure (New York Heart Association 3 to 4 or ejection fraction <40%), or untreated bilateral carotid stenosis (>70%). OXVASC is approved by the Oxfordshire Research Ethics Committee. Requests for access to the data and analysis tools in this article will be openly considered. Please contact the chief investigator of OXVASC for further information (peter.rothwell@ndcn.ox.ac.uk).

### Assessment of CVR

CVR was assessed by BH and hyperventilation. Patients underwent continuous, noninvasive BP monitoring (Finometer MIDI; Finapres Medical Systems), end-tidal CO_2_ (etCO_2_) via nasal cannulae (Capnocheck Plus; Smith Medical), ECG at 200 Hz (Finometer MIDI), and bilateral middle cerebral artery blood flow velocity. The middle cerebral artery was ideally defined by the peak flow velocity identifiable at the nearest depth to 50 mm at an appropriate insonation angle, in a vessel with flow toward the probe extending for at least 0.5 cm.

After establishing reliable monitoring, patients remained at supine rest for 10 minutes. They were instructed how to perform the BH procedure and then asked to hold their breath at the end of a normal volume inspiration for 30 seconds. At the end of 30 seconds, or if the patient was unable to maintain apnea, they were asked to exhale via their nose to end expiration. If an inadequate BH was achieved, or if there was a significant Valsalva effect (mean blood pressure [MBP] decrease of >10 mm Hg in the first half of the BH), the patient was instructed again on how to perform the maneuvre, concentrating on taking a normal tidal volume inspiration, and the procedure was repeated. After normalization of cerebral blood flow and a minimum of 1 minute of rest, the patient was instructed as to how to hyperventilate. They were then asked to hyperventilate via their nose for 30 seconds at a normal tidal volume and a rate of 30 to 60 breaths per minute. If the fall in etCO_2_ was inadequate, the patient was asked to increase their respiratory rate or tidal volume to target a minimum 10% fall in etCO_2_. The test could be repeated once if inadequate.

### Analysis

All data were acquired in Labchart v8 and analyzed with dedicated in-house software (Matlab). Systolic BP, diastolic BP, MBP, etCO_2_ and peak, trough, and mean flow velocity (MFV) on transcranial Doppler (TCD) were extracted, with automated detection of baseline values (mean over 5 seconds before apnea or hyperventilation) and minimum or maximum values at end of respiratory maneuvres and during the first 30 seconds of recovery. All records were visually reviewed and manually corrected by 2 independent assessors blind to clinical information and scored for quality of recording (unusable, severe artifact and likely inaccurate, adequate quality, high quality, optimal quality). Only records with adequate to optimal quality of BP, capnography, and at least 1 TCD side were included. For TCD analysis, the side with the better recording quality on blinded assessment was used; if both sides were considered of equal quality, the right side was used.

Change in BP, flow velocity, or etCO_2_ was calculated as the absolute increase (BH) or decrease (hyperventilation) from baseline to the greatest absolute change, and as the change in MFV per mm Hg change in etCO_2_, expressed as absolute values or percentage change from baseline. Associations between change in each variable and age or demographics were determined by linear regression for continuous variables or *t* tests for binary variables. Agreement between left and right MCA was visualized by Bland-Altman plots.

Associations between demographics, etCO_2_, and BP with change in MFV, peak systolic velocity (PSV), or end-diastolic velocity (EDV) were determined by general linear models for change in etCO_2_ and change in systolic BP, diastolic BP, or MBP, unadjusted and adjusted for age and sex. A nonlinear interaction between ΔBP and ΔetCO_2_ was empirically determined by simulation with sequential inclusion of individuals by ΔBP for a threshold above and below which there was an independent association between ΔetCO_2_ and ΔMFV. General linear models were repeated with stratification of independent variables (ΔBP, ΔetCO_2_) at this empirically determined threshold and across quartiles.

*P*<0.05 was taken as significant. Analyses were performed in IBM SPSS v25, Matlab r2015, or Windows Excel.

## Results

Four hundred eighty-eight of 602 patients with adequate bone windows (81%) had good-quality recordings during BH, of whom 426 had good-quality hyperventilation tests. Good-quality recordings were more common in younger participants (64.6 versus 68.7 years; *P*<0.005), with no significant differences in demographics or baseline TCD measures between patients with only BH, only hyperventilation, or both tests (Table 1). For patients with bilateral recordings, there was good agreement between sides, with no significant evidence of bias on regression of Bland-Altman plots (BH: r=0.129, R^2^=0.17, *P*=0.087; hyperventilation: r=0.039, R^2^=0.002, *P*=0.606; Figure I in the online-only Data Supplement).

**Table 1.**
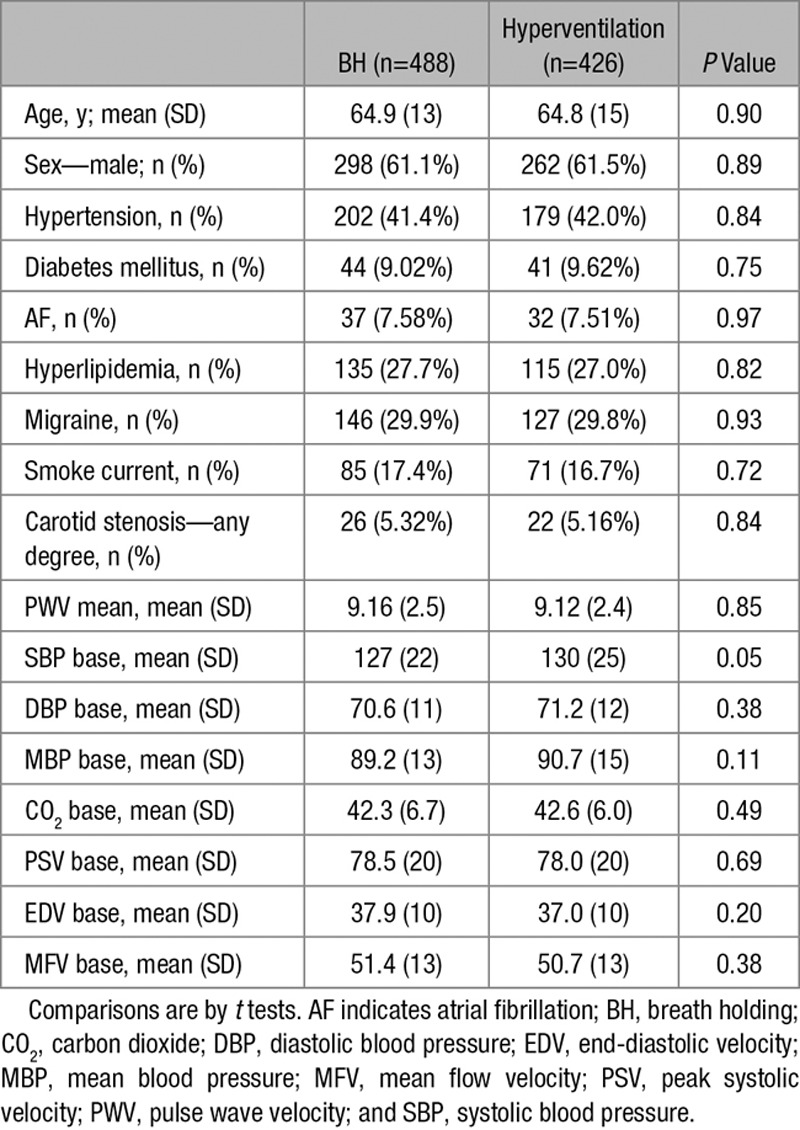
Clinical Characteristics of Patients With Adequate Quality Assessments of Cerebrovascular Reactivity

Baseline BP, MFV, and etCO_2_ were negatively correlated with age, while women had lower etCO_2_ and MFV. Patients with hypertension had lower baseline MFV, whereas current smokers had higher baseline MFV (Table 2). However, there was no correlation between baseline etCO_2_ and MFV (r=0.089, *P*=0.051) and between baseline MBP and MFV (r=−0.027, *P*=0.559).

**Table 2.**
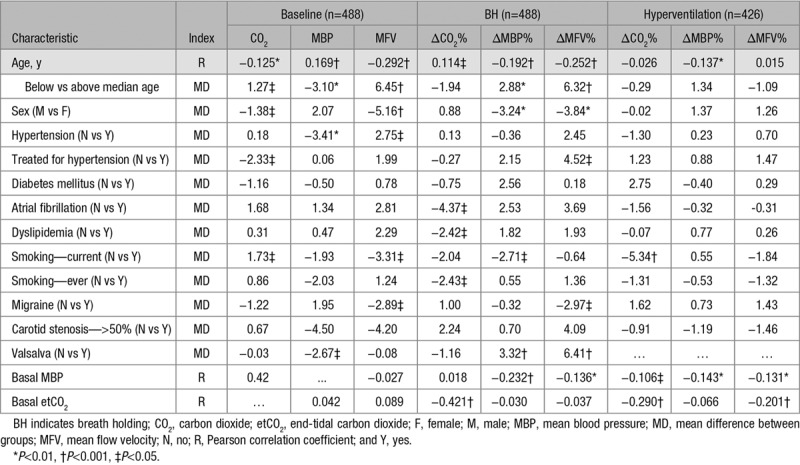
Associations Between Change in CO_2_, MBP, and MFV With Clinical Characteristics

Age was associated with a smaller increase in MFV and MBP but a larger increase in etCO_2_ during BH but during hyperventilation was only associated with a greater fall in MBP, with no association with change in MFV or etCO_2_ (Table 2). In contrast, MBP and MFV increased more in women during BH, whereas there was no significant difference between sexes in response to hyperventilation. Use of antihypertensives was associated with a lesser increase in MFV during BH, despite only a nonsignificant relationship with decrease in MBP, with no significant change during hyperventilation (Table 2). In contrast, although atrial fibrillation and smoking were associated with significant differences in change in etCO_2_, they were not associated with any difference in change in MFV.

### Relationship Between BP, CO_2_, and Cerebral Blood Flow

**Figure 1.**
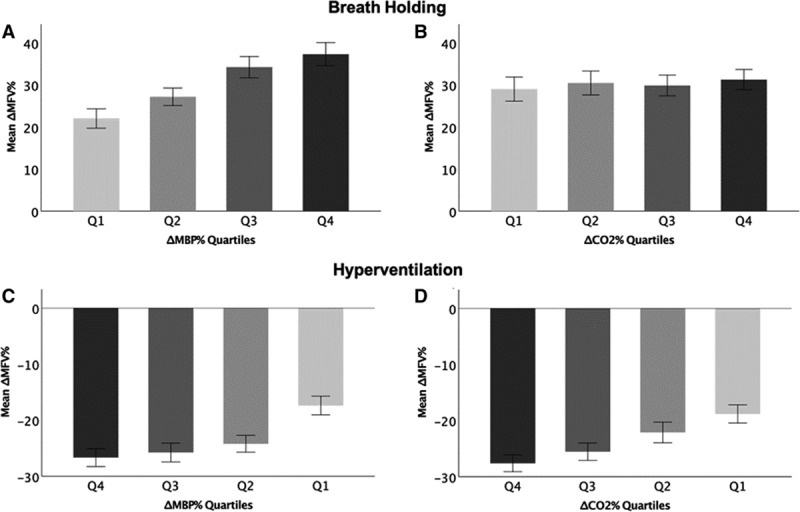
Percentage change in mean flow velocity (MFV) during breath holding or hyperventilation by quartile of change in mean blood pressure (MBP) or end-tidal carbon dioxide (CO_2_). **A** and **B** show change in MFV during breath holding by quartile of change in MBP (**A**) and CO_2_ (**B**). **C** and **D** show ΔMBP% during hyperventilation by ΔMBP (**C**) or ΔCO_2_ (**D**), with magnitude of change increasing from Q1 to Q4. Data show mean change with 95% CIs as error bars.

During BH, there was a significant increase in MFV, which was correlated with a concurrent increase in MBP (r^2^=0.15, *P*<0.001; Table 3) but was not correlated with the increase in CO_2_ (r^2^=0.002, *P*=0.32). However, during hyperventilation, the fall in MFV was correlated with both reduction in CO_2_ and reduction in MBP (Figure 1), with a similar strength of association between both CO_2_ and MBP with MFV. These associations persisted after adjustment for age and sex (Table 3) and when assessing percentage change from baseline (Table 3) or absolute change (Table I in the online-only Data Supplement), with an average 0.3% increase in MFV per 1% increase in etCO_2_ during hyperventilation, consistent before and after adjustment, and across strata of change in BP.

**Table 3.**
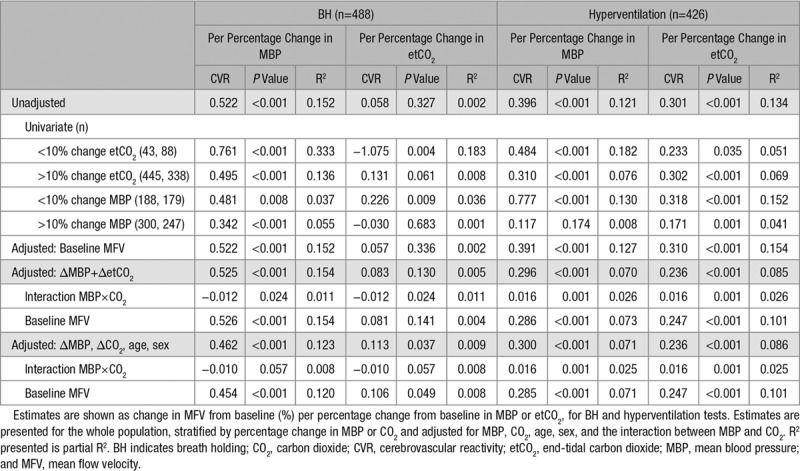
Middle Cerebral Artery Reactivity to Changes in Blood Pressure or CO_2_

Change in MFV was significantly associated with both changes in PSV and EDV for both respiratory tests. Although with both tests there were similar associations between systolic BP with PSV and diastolic BP with EDV (Tables II and III in the online-only Data Supplement), there was no relationship during BH between change in etCO_2_ and either PSV or EDV. In contrast, during hyperventilation, etCO_2_ was associated with change in both PSV and EDV.

Although there was no significant linear interaction between change in CO_2_ and change in MBP with change in MFV during BH, there was a nonlinear interaction on empirical modeling (Figure II in the online-only Data Supplement); conversely, there was a significant linear interaction between change in CO_2_ and change in MBP during hyperventilation. The association between change in etCO_2_ with change in MFV with sequentially increasing levels of change in MBP demonstrated a threshold effect for a significant association at an ≈10% change in MBP during both respiratory tests (Figure III in the online-only Data Supplement). In the subgroup of patients with a change in MBP of <10% during BH, there was a significant association between change in etCO_2_ and change in MFV, with no association in patients with a change in MBP >10% (Table 3). Similarly, there was a stronger association between change in MBP and change in MFV in patients with change in CO_2_ <10%.

During hyperventilation, the relationship between change in etCO_2_ and change in MFV was slightly greater in patients with a change in MBP <10%, but it remained significant in patients with a change in MBP >10%, while there was a stronger relationship between MBP and MFV in patients with a change in CO_2_ <10% (Table 3).

Although 53.1% (259 of 488) of patients performed an involuntary Valsalva maneuvre during BH, despite repeated instruction, there was no difference in the strength of associations with change in MFV in patients performing a Valsalva for either CO_2_ (*P*=0.151 and *P*=0.631, respectively) or MBP (r^2^=0.147, *P*<0.0005, versus r^2^=0.128, *P*<0.0005), despite a lower average change in MFV (Table 2). Baseline MBP was associated with ΔMFV during both BH and hyperventilation, but baseline etCO_2_ was only associated with ΔMFV during hyperventilation. This reflected the association between baseline values and ΔMBP and ΔetCO_2_, respectively (Table 2). Across quartiles of baseline MBP during hyperventilation, ΔMBP decreased with a corresponding smaller decrease in MFV (Figure IV in the online-only Data Supplement). There were no significant associations between the cerebrovascular responses to hyperventilation versus BH, either for percentage change in MFV (r^2^=0.006, *P*=0.09) or percentage change in MFV per percentage change in CO_2_ (r^2^=0.0006, *P*=0.61), although there was a weak association between absolute changes in MFV on the 2 respiratory maneuvres (r^2^=0.03, *P*=0.0003), reflecting the overall magnitude of MFV.

**Figure 2.**
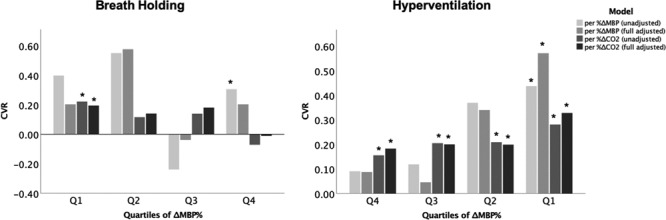
Magnitude of cerebrovascular reactivity (CVR) during breath holding and hyperventilation by quartile of change in mean blood pressure (MBP). CVR (percentage change in mean flow velocity [MFV] per percentage change in MBP or carbon dioxide [CO_2_]) is derived from general linear models, unadjusted and adjusted for age, sex, and baseline MFV. *P<0.05.

On stratifying change in MBP or etCO_2_ into quartiles, ΔMFV increased linearly across all quartiles of ΔMBP during BH, with no difference in ΔMFV across quartiles of ΔetCO_2_. However, there was a linear decrease in ΔMFV across quartiles of ΔetCO_2_ during hyperventilation (Figure 1). Similarly, the magnitude of the relationship between ΔetCO_2_ and ΔMFV varied by quartile of ΔMBP during BH, before and after adjustment for confounders, with the only significant association in the lowest quartile of ΔMBP (Figure 2). However, during hyperventilation, the magnitude of association (the CVR) between ΔetCO_2_ and ΔMFV was consistent across quartiles of ΔMBP, before and after adjustment for baseline MFV and age and sex.

## Discussion

In the prospective OXVASC phenotyped cohort of patients with recent transient ischemic attack or minor stroke, cerebral blood flow responses to apnea were principally determined by increases in systemic BP, while etCO_2_ only induced changes when BP change was <10%. In contrast, change in MFV during hyperventilation was determined by both change in systemic BP and change in etCO_2_, with a consistent relationship with etCO_2_ across different levels of change in BP and demographic characteristics. Findings were consistent for change in systolic or diastolic measures and for absolute and percentage changes.

Previous studies identified a reduction in CVR in patients with lacunar stroke,^23^ white matter hyperintensities,^5^ and cognitive impairment,^15^ but a direct causative role for endothelial dysfunction is unproven.^23^ Yet, with evidence of blood-brain barrier breakdown in small vessel disease,^24^ a number of studies are targeting CVR to prevention of progression of small vessel disease (LACI [Laser Angioplasty for Critical Limb Ischemia], TREAT-SVDs [Effects of Amlodipine and Other Blood Pressure Lowering Agents on Microvascular Function in Small Vessel Diseases], OxHARP [Oxford Haemodynamic Adaptation to Reduce Pulsatility Trial]). A reduction in CVR has also been found in hemodynamically significant carotid stenosis, likely reflecting maximal cerebral vasodilatation and reduced cerebrovascular reserve.^10,11^ This finding is associated with symptomatic rather than asymptomatic carotid stenosis,^14^ severity of stenosis, and an increased risk of recurrent stroke.^11^

Despite the potential clinical significance of CVR, there is no standardized method of CVR measurement, either for measuring blood flow or for inducing change in CO_2_. Increased cerebral perfusion on magnetic resonance imaging with inhaled CO_2_ is the current preferred test, but this requires expertise, equipment, and time; cannot be performed with concurrent BP measurement; is poorly tolerated by many patients; and is not feasible in frail, elderly patients, and its dependence on BP changes is unknown. Alternative CVR challenges have been used in larger populations, measuring change in mean cerebral blood flow (MFV) with transcranial Doppler ultrasound or near-infrared spectroscopy to induced CO_2_ changes through BH, hyperventilation, acetazolamide, or inhaled CO_2_. All methods assume that the change in cerebral blood flow reflects the endothelium-dependent response to CO_2_ but do not determine the potential contribution of concurrent change in BP. In limited studies, changes in BP driving cerebral blood flow changes have been identified in small studies with BH,^25–27^ hyperventilation,^28^ and CO_2_ rebreathing,^28^ but the effect of the magnitude of BP changes on the validity of different tests is unclear.

Our study demonstrated that cerebral blood flow responses to both BH and hyperventilation reflected changes in BP and CO_2_, but the response during BH was dominated solely by BP, except in limited patients in whom no BP response was seen. This was not dependent on the presence of a Valsalva and occurred despite efforts to train the participant, likely reflecting endogenous sympathetically driven BP rises to the stress of apnea.^27^ However, during hyperventilation, the response was consistently dependent on both BP change and CO_2_. Although this affects the interpretation of the result for an individual, this allows for reliable interpretation of the importance of BP and CO_2_-driven responses across a population where both indices are measured. Even in an individual, CO_2_-dependent CVR could be estimated by adjusting for the expected change in MFV because of BP change. The greater validity of hyperventilation is supported by its reduced association with age, consistent with studies reporting greater reproducibility and preserved cerebrovascular function in healthy aging.^29^ We did not study change in BP during inhaled CO_2_, but these tests are innately stressful with ≤5% of patients not tolerating CO_2_ inhalation during magnetic resonance imaging and may well be associated with sympathetically driven rises in BP that would confound interpretation of CVR in the absence of BP measurement. This does not undermine the interpretation that CVR impairment assessed by BH in carotid stenosis is prognostically significant but undermines the interpretation that abnormalities in CVR in small vessel disease reflect cerebrovascular endothelial dysfunction and that CVR as opposed to autoregulation to BP is the optimal treatment target.

There are limitations to our study. First, the study was performed in consecutive, unselected patients with transient ischemic attack or minor stroke, resulting in a high proportion of elderly and frail patients who may have had greater difficulties in performing the maneuvres, and a relatively high proportion of patients with poor TCD windows or poor quality recordings. However, the analysis was restricted to good-quality recordings, and this population reflects the population of interest for assessment of CVR. Second, we did not assess the effects of BP during CO_2_ inhalation or during magnetic resonance imaging–based methods of cerebral blood flow assessment. This limits extrapolation of these results to these tests, which require further study to determine whether they are similarly confounded by concurrent BP changes. Third, although the OXVASC study and the physiological tests were prespecified in the protocol of the study, the details of the analysis were not. Fourth, the time constraints of performing a complex series of physiological tests in a pragmatic clinical population resulted in a maximum of 2 repeats of each test. However, this study, therefore, reflects an assessment of the validity of these tests used within a busy clinical setting. Finally, because of practical limitations in an elderly, population-based cohort, the respiratory maneuvres did not include techniques to control the magnitude of etCO_2_ change, such as paced breathing, end-tidal forcing, or biofeedback. However, this was not practical without limiting inclusivity, and etCO_2_ change was linearly associated with MFV during hyperventilation and sufficiently large in most patients to allow for reliable estimation of the effect of etCO_2_ change.

Overall, this study identified that hyperventilation is a reliable and practical method to assess CVR in a large population with acute cerebrovascular disease, but concurrent measurement of continuous BP response is critical for validly interpreting the results. However, BH principally reflects the cerebrovascular response to BP change in this population. Although this may still have prognostic value in a large population and may reflect autoregulation of cerebral blood flow responses to BP, concurrent BP measurement is critical and the measured outcome cannot be interpreted as reflecting endothelium-dependent CO_2_ responses. Further research is required to assess the role of BP changes in CVR assessment during inhaled CO_2_, to assess the prognostic significance of BH and hyperventilation, to determine the CVR-dependent effects of antihypertensive and statin-based treatments,^30^ for stroke prevention, and to address which determinants of the cerebrovascular response are associated with cerebral small vessel disease.

## Acknowledgments

We are grateful to the staff in the general practices that collaborated in the OXVASC (Oxford Vascular Study): Abingdon Surgery, Stert St, Abingdon; Malthouse Surgery, Abingdon; Marcham Road Family Health Centre, Abingdon; The Health Centre, Berinsfield; Key Medical Practice; Kidlington; 19 Beaumont St, Oxford; East Oxford Health Centre, Oxford; Church Street Practice, Wantage. This work uses data provided by patients and collected by the National Health Service as part of their care and support and would not have been possible without access to these data. A.J.S. Webb devised, acquired, supervised, and analyzed physiological assessments and statistical analysis and drafted, edited, and submitted the manuscript. Dr Paolucci acquired and analyzed physiological data, performed statistical analyses, and had equal responsibility for the manuscript. Dr Mazzucco and L. Li acquired physiological assessments. P.M. Rothwell established and supervised the OXVASC study and devised, initiated, and supervised the physiological studies, analyses, and manuscript.

## Sources of Funding

OXVASC (Oxford Vascular Study) is funded by the National Institute for Health Research (NIHR) Oxford Biomedical Research Centre, Wellcome Trust, Wolfson Foundation, British Heart Foundation, and the European Union’s Horizon 2020 programme (grant 666881, SVDs@target). P.M. Rothwell is in receipt of an NIHR Senior Investigator award. A.J.S. Webb is funded by a Wellcome Trust clinical research career development Fellowship (206589/Z/17/Z) and British Heart Foundation Project Grant (PG/16/38/32080).

## Disclosures

None.

## Supplementary Material
