# Supplementary material for: Confounding of Cerebral Blood Flow Velocity by Blood Pressure During Breath Holding or Hyperventilation in Transient Ischemic Attack or Stroke
Source: Stroke. 2020 Jan 27;51(2):468–74. doi: 10.1161/STROKEAHA.119.027829 (PMC7004447; doi:10.1161/STROKEAHA.119.027829)

SUPPLEMENTAL MATERIAL

Confounding of cerebral blood flow velocity by blood pressure during breath-holding or hyperventilation in TIA or stroke

Webb AJS*†, Paolucci M*±, Mazzucco S†, Li L†, Rothwell PM,† for the Oxford Vascular Study Phenotyped Cohort

Supplementary Figure I. Bland-Altman plots for agreement in ∆MFV comparing left and right MCA velocities during breath-holding and hyperventilation.

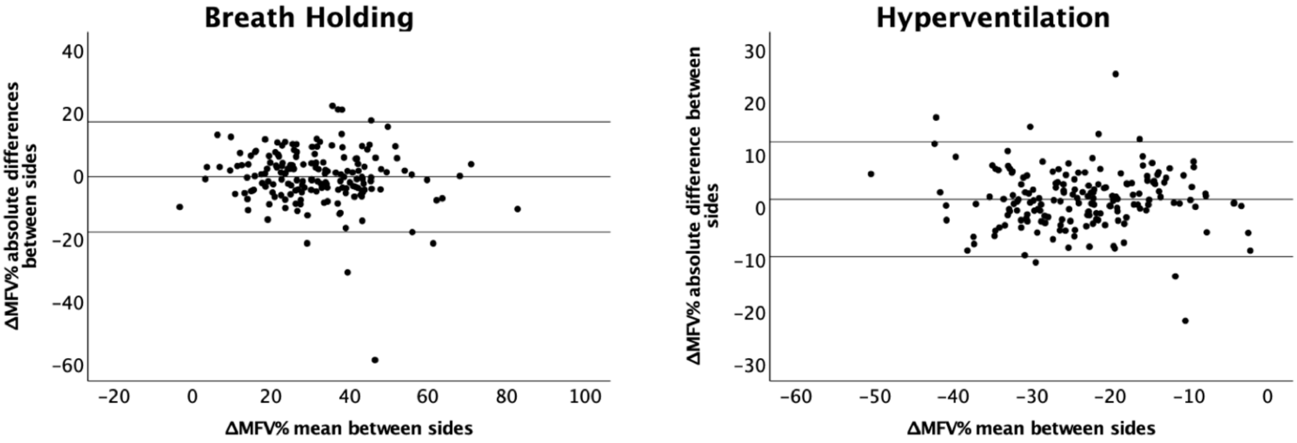

Supplementary Figure II Linear associations between change in MBP or etCO2 with MFV during breath-holding (A + B) and hyperventilation (C + D).

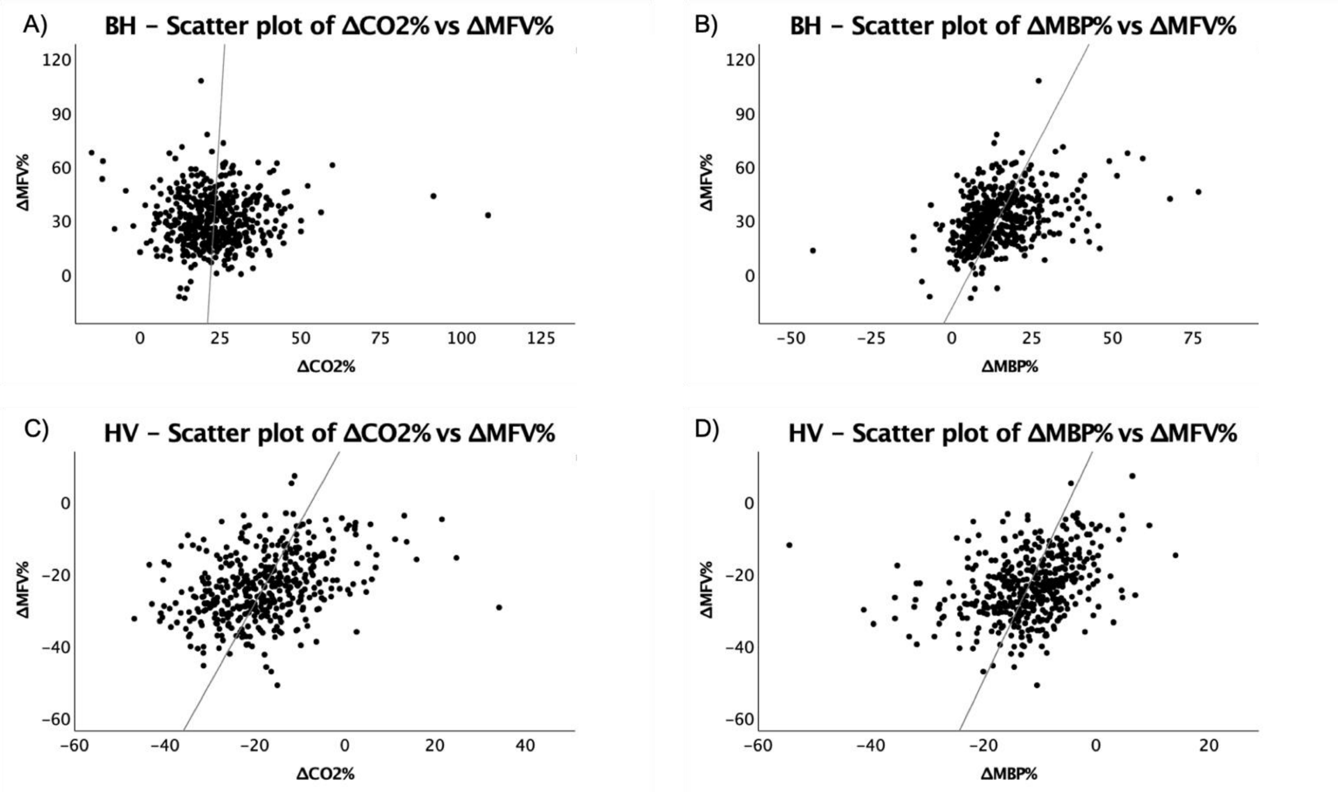

Supplementary Figure III. Strength of association (r^2) between change in CO2 and change in MFV during breath-holding by increasing percentage change in MBP. Results are shown for the linear association for increasingly large populations, consecutively including patients by increasing level of change in MBP. A threshold for a significant association between change in CO2 and MFV is found below an approximately 10% change in MBP.

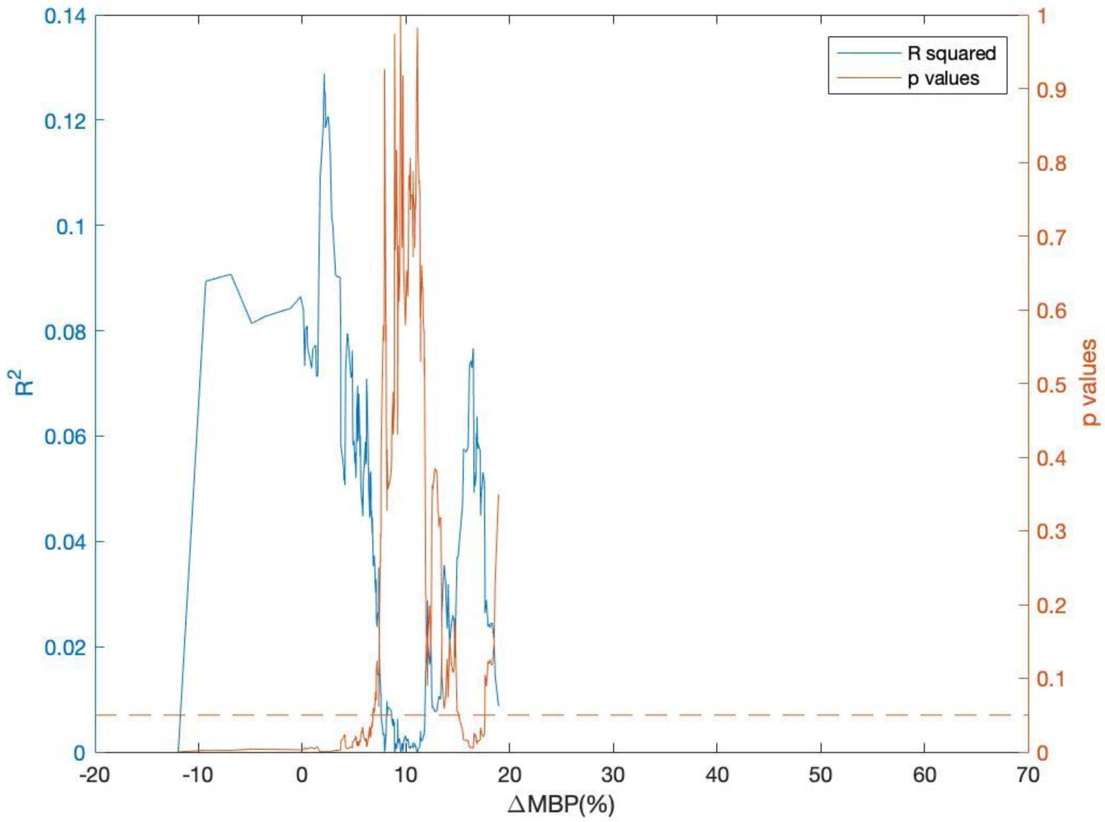

Supplementary table I – Middle cerebral artery reactivity to changes in blood pressure or carbon dioxide. Estimates are shown as change in mean flow velocity from baseline (m/s) per absolute change from baseline in mean blood pressure (MBP, mmHg) or end-tidal carbon dioxide (etCO2, % of atmospheric pressure), for breath-holding and hyperventilation tests. Estimates are presented for the whole population; for patients with greater or less than 10% change in etCO2 or MBP; adjusted for MBP or CO2, interaction between MBP and CO2, baseline mean flow velocity and for age and gender. R^2 presented are partial R^2.

	Breath-Holding (n=488)						Hyperventilation (n=426)					
	Per mmHg change in MBP			Per % change in etCO2			Per mmHg change in MBP			Per % change in etCO2		
	CVR	p-value	R^2	CVR	p-value	R^2	CVR	p-value	R^2	CVR	p-value	R^2
Unadjusted	.312	< .0005	.108	0.27	.762	.000	.207	< .0005	.067	.342	< .0005	.075
Univariate												
<10% change etCO2	.382	< .0005	.259	-1.038	.019	.126	.308	< .0005	.161	.395	.052	.043
>10% change etCO2	.303	< .0005	.096	.067	.600	.001	.155	< .0005	.036	.338	< .0005	.045
<10% change MBP	.308	.011	.034	.195	.150	.011	.503	< .0005	.101	.450	< .0005	.135
>10% change MBP	.155	.012	.021	-.024	.828	.000	.063	.279	.005	.137	.109	.010
Adjusted: Baseline MFV	.305	< .0005	.125	.064	.434	.001	.198	< .0005	.116	.350	< .0005	.149
Adjusted: ∆MBP + ∆etCO2	.314	< .0005	.109	.070	.411	.001	.156	< .0005	.037	.271	< .0005	.046
Interaction MBP * CO2	-.007	.480	.001	-.007	.480	.001	.022	.003	.021	.022	.003	.021
and baseline MFV	.308	< .0005	.128	.106	.169	.004	.145	< .0005	.066	.284	< .0005	.102
Adjusted: MBP, ∆CO2, Age, Sex	.254	< .0005	.082	.080	.316	.002	.171	< .0005	.045	.263	< .0005	.045
Interaction MBP * CO2	-.003	.713	.000	-.003	.713	.000	.022	.003	.021	.022	.003	.021
and baseline MFV	.270	< .0005	.104	.108	.148	.004	.144	< .0005	.064	.284	< .0005	.101

Supplementary table II – Middle cerebral artery reactivity to changes in blood pressure or carbon dioxide. Estimates are shown as change in peak flow velocity from baseline (m/s) per absolute change from baseline in systolic blood pressure (SBP, mmHg) or end-tidal carbon dioxide (etCO2, % of atmospheric pressure), for breath-holding and hyperventilation tests. Estimates are presented for the whole population; for patients with greater or less than 10% change in etCO2 or MBP; adjusted for SBP or CO2, interaction between SBP and CO2, baseline mean flow velocity and for age and gender. R^2 presented are partial R^2.

	Breath-Holding (n=488)						Hyperventilation (n=426)					
	Per mmHg change in SBP			Per % change in etCO2			Per mmHg change in SBP			Per % change in etCO2		
	CVR	p-value	R^2	CVR	p-value	R^2	CVR	p-value	R^2	CVR	p-value	R^2
Unadjusted	.246	< .0005	.089	.096	.385	.002	.188	< .0005	.070	.424	< .0005	.060
Univariate												
<10% change etCO2	.250	.038	.100	-1.574	.003	.197	.285	< .0005	.201	.362	.183	.021
>10% change etCO2	.245	< .0005	.087	.179	.245	.003	.140	< .0005	.036	.424	< .0005	.036
<10% change MBP	.048	.628	.001	.254	.135	.012	.381	< .0005	.084	.502	< .0005	.089
>10% change MBP	.146	.005	.026	.055	.681	.001	.104	.039	.017	.218	.067	.014
Adjusted: Baseline MFV	.247	< .0005	.102	.134	.195	.003	.184	< .0005	.110	.433	< .0005	.104
Adjusted: ΔSBP + ΔetCO2	.248	< .0005	.090	.117	.265	.003	.150	< .0005	.043	.320	< .0005	.034
Interaction SBP * CO2	.006	.512	.001	.006	.512	.001	.012	.077	.007	.012	.077	.007
and baseline MFV	.249	< .0005	.104	.156	.111	.005	.144	< .0005	.070	.333	< .0005	.064
Adjusted: SBP, ΔCO2, Age, Sex	.214	< .0005	.073	.125	.216	.003	.155	< .0005	.044	.312	< .0005	.034
Interaction SBP * CO2	.010	.262	.003	.010	.262	.003	.011	.082	.007	.011	.082	.007
and baseline MFV	.228	< .0005	.089	.154	.110	.005	.129	< .0005	.052	.341	< .0005	.067

Supplementary table III – Middle cerebral artery reactivity to changes in blood pressure or carbon dioxide. Estimates are shown as change in end-diastolic flow velocity from baseline (m/s) per absolute change from baseline in diastolic blood pressure (DBP, mmHg) or end-tidal carbon dioxide (etCO2, % of atmospheric pressure), for breath-holding and hyperventilation tests. Estimates are presented for the whole population; for patients with greater or less than 10% change in etCO2 or MBP; adjusted for DBP or CO2, interaction between DBP and CO2, baseline mean flow velocity and for age and gender. R^2 presented are partial R^2.

	Breath-Holding (n=488)						Hyperventilation (n=426)					
	Per mmHg change in DBP			Per % change in etCO2			Per mmHg change in DBP			Per % change in etCO2		
	CVR	p-value	R^2	CVR	p-value	R^2	CVR	p-value	R^2	CVR	p-value	R^2
Unadjusted	.340	< .0005	.110	-.011	.896	.000	.215	< .0005	.052	.299	< .0005	.066
Univariate												
<10% change etCO2	.426	< .0005	.313	-.680	.099	.065	.309	.001	.118	.411	.024	.058
>10% change etCO2	.329	< .0005	.097	.000	.999	.000	.166	.001	.030	.288	< .0005	.036
<10% change MBP	.499	< .0005	.095	.155	.223	.008	.364	.006	.043	.423	< .0005	.119
>10% change MBP	.164	.014	.020	-.063	.551	.001	.068	.284	.005	.097	.206	.007
Adjusted: Baseline MFV	.327	< .0005	.126	.027	.728	.000	.203	< .0005	.079	.308	< .0005	.118
Adjusted: ∆DBP + ∆etCO2	.342	< .0005	.111	.044	.585	.001	.160	< .0005	.029	.246	< .0005	.043
Interaction DBP * CO2	-.014	.156	.004	-.014	.156	.004	.026	.004	.020	.026	.004	.020
and baseline MFV	.330	< .0005	.128	.080	.271	.003	.147	< .0005	.043	.258	< .0005	.084
Adjusted: DBP, ∆CO2, Age, Sex	.263	< .0005	.078	.052	.484	.001	.169	< .0005	.033	.243	< .0005	.044
Interaction DBP * CO2	-.011	.205	.003	-.011	.205	.003	.026	.003	.021	.026	.003	.021
and baseline MFV	.280	< .0005	.100	.081	.243	.003	.151	< .0005	.046	.258	< .0005	.084

Supplementary Figure IV. **Mean change in percentage MFV or MBP during breath-holding and hyperventilation across quartiles of change in baseline MBP.**

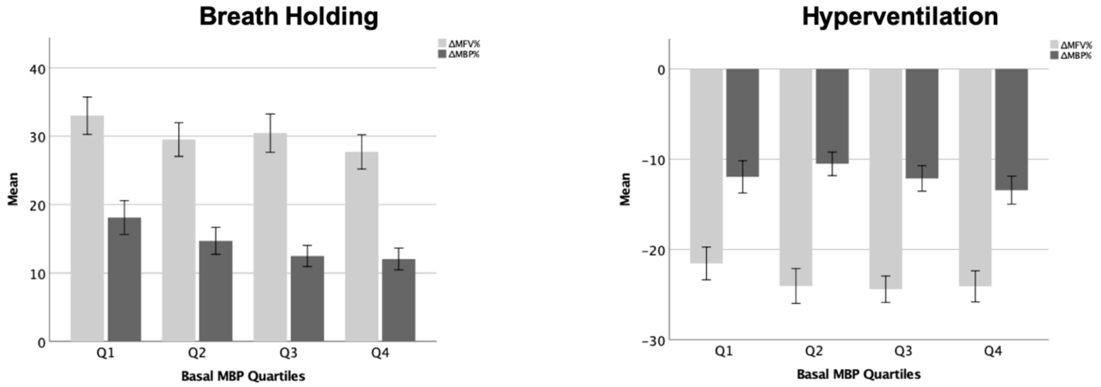

Supplement: Supplementary file 1 [file str-51-468-s001.pdf]
